# The Use of Bacteriophages in Biotechnology and Recent Insights into Proteomics

**DOI:** 10.3390/antibiotics11050653

**Published:** 2022-05-13

**Authors:** Ana G. Abril, Mónica Carrera, Vicente Notario, Ángeles Sánchez-Pérez, Tomás G. Villa

**Affiliations:** 1 Department of Microbiology and Parasitology, Faculty of Pharmacy, University of Santiago de Compostela, 15898 Santiago de Compostela, Spain; ana.gonzalez.abril@usc.es; 2 Department of Food Technology, Spanish National Research Council (CSIC), Marine Research Institute (IIM), 36208 Vigo, Spain; mcarrera@iim.csic.es; 3 Lombardi Comprehensive Cancer Center, Georgetown University, Washington, DC 20057, USA; notariov@georgetown.edu; 4 Sydney School of Veterinary Science, Faculty of Science, University of Sydney, Sydney, NSW 2006, Australia; angelines2085@icloud.com

**Keywords:** phage-based proteomics, LC–ESI–MS/MS, mass spectrometry, bacteriophage, bacterial detection, antimicrobials, vaccines

## Abstract

Phages have certain features, such as their ability to form protein–protein interactions, that make them good candidates for use in a variety of beneficial applications, such as in human or animal health, industry, food science, food safety, and agriculture. It is essential to identify and characterize the proteins produced by particular phages in order to use these viruses in a variety of functional processes, such as bacterial detection, as vehicles for drug delivery, in vaccine development, and to combat multidrug resistant bacterial infections. Furthermore, phages can also play a major role in the design of a variety of cheap and stable sensors as well as in diagnostic assays that can either specifically identify specific compounds or detect bacteria. This article reviews recently developed phage-based techniques, such as the use of recombinant tempered phages, phage display and phage amplification-based detection. It also encompasses the application of phages as capture elements, biosensors and bioreceptors, with a special emphasis on novel bacteriophage-based mass spectrometry (MS) applications.

## 1. Introduction

Bacteriophages, or simply phages, are viruses of prokaryotes that represent the most abundant organisms on Earth. Phages were discovered in the early years of the 20th century and originally recognized for their antibacterial activity [1]. In addition, both bacteriophages and bacteria are the most abundant and diverse entities found in our planet, as they both display considerable genetic and phenotypic variability due to their high mutation rates and short generation times. The interaction between phages and bacteria is the result of an evolutionary co-adaptation, resulting in a fast and dynamic co-evolution which, in some cases, facilitates the conversion of harmless bacteria into pathogenic organisms [2]. The integration of the genome of temperate bacteriophages into the chromosomes of bacteria can either be beneficial to the bacterial host, when leading to the acquisition of novel functions, or detrimental, when insertional events interrupt a gene and/or alter bacterial gene expression [3]. Phage-mediated transduction allows genes to be transferred from a donor bacterium to a recipient microorganism, hence providing an additional mechanism by which bacteria can gain novel genes. Gene excisions and integrations are mediated by DNA recombinase, an enzyme produced by phages [4,5]. Horizontal Gene Transfer (HGT) is the main process responsible for intra-species genomic differences, hence bacteriophages are probably the major contributors to bacterial genome diversification; additional gene transfer mechanisms include integrative plasmids and transposons [5,6]. The co-evolution of phages and bacteria was demonstrated by the presence of virulence factors, originally from bacteriophages, in bacterial cells, these factors allow the microorganisms to infect mammalian cells. Virulence factors include toxins as well as molecules that promote adhesion, colonization, resistance, or immune system evasion; they also involve transcription factors that regulate bacterial genes [7]. Phages, and the proteins they encode, have been used in the development of a variety of diagnostic assays that allow specific molecule identification and even bacterial detection. The fact that bacteriophages are specific to particular bacteria and can only infect certain bacterial hosts, together with the shared co-evolution between phage and host (i.e., determination of specific phage biomarkers), make the viruses good tools to facilitate the identification of, otherwise, hard to detect bacteria.

Bacteriophage research has recently undergone a revival, due to the ominous threat to global human health posed by antibiotic resistance. This revitalization has resulted in a series of improvements in areas including high-resolution microscopy, DNA manipulation, and sequencing technologies. The mobilized colistin resistance gene *mcr-1*, which confers resistance to colistin (one of the last-resort antibiotics to treat infections caused by Gram-negative bacteria), was described in an *E. coli* strain in a pig in 2015. Since then, this gene has been identified in several countries around the world, as well as in a wide variety of bacterial species belonging to genera such as *Escherichia*, *Salmonella*, *Klebsiella*, *Citrobacter*, and *Cronobacter*. Phages infecting bacterial species included in these genera, have been isolated, their proteins identified, and their functions deciphered. Consequently, the roles played by these phage proteins, either in the bacteriophage cycle or in bacterial genetics are currently known. These bacteriophages, as well as other phages that infect antibiotic-resistant pathogens, represent putative essential tools in the fight against multidrug resistant bacterial infections, either on their own or in combination with antibiotics. In addition, these phages can also play a major role in the design of specific, affordable, and stable sensors for the detection of specific bacteria [8]. The intrinsic properties of bacteriophages extend the putative use of these organisms in multiple scientific fields, including health, industry, food science, agriculture, and food safety, as well as in the molecular typing, control, and detection of bacteria. Identification of phage-encoded proteins and understanding of their functions, also open the way for the potential use of bacteriophages as vehicles for drug delivery and vaccine development.

This review summarizes the use of a variety of phage-based techniques, from methods to identify specific bacteria to the utilization of mass spectrometry (MS) applications to rapidly analyze and characterize phage proteins.

## 2. Exploiting Bacteriophage Proteomes

Recent improvements in phage sequencing, DNA manipulation and synthetic biology have led to an escalation in phage proteome discovery, paving the way to multitude of potential applications in a wide variety of scientific fields. Nevertheless, despite the extensive current knowledge about phage-encoded proteins, there are still many of these polypeptides with yet unknown functions.

The key to expand the commercial uses of bacteriophages resides in understanding the phage replication cycle and identifying the biotechnological potential of the phage-coded proteins, taking also into consideration that some of the viral proteins may be used in a variety of applications [9]. What follows is a summary of some of the most important phage proteins so far identified.

Receptor binding proteins (RBPs): These viral proteins are part of the bacteriophage particle and are involved in host-phage interaction. As they are responsible for bacterial host identification, these polypeptides can be utilized in specific bacterial pathogen detection, diagnosis, and therapy. The RBPs are specific for, and can distinguish between, glycosylation variants of O-antigens [10], which constitute the outermost portion of the lipopolysaccharide (LPS) covering the surface of Gram-negative bacteria. In fact, the binding affinity and specificity of RBPs have been successfully used in the production of therapeutic proteins as well as in processes designed to remove important and/or dangerous bacterial contaminants, such as bacterial endotoxin [9,11].

Depolymerases: The bacterial capsule provides protection against a variety of factors that are detrimental to the bacterium, such as host immunity, antibiotics, and desiccation [12]. It also potentiates adherence to host cells and surfaces and protects bacterial cells from phage infection. However, some bacteriophages use the capsule as an adsorption receptor, degrading the capsular polysaccharides (CPS) and penetrating this bacterial outer layer to get access to their receptors on the cell membrane [13]. These steps are essential for bacteriophages to be able to inject their nucleic acid inside the bacterial cell. The phage enzymes responsible for degrading the CPS are known as depolymerases and can either be found as part of the phage structure or be released into the media as free enzymes. Depolymerases are multifaceted proteins that can be used in a variety of applications. They specifically hydrolyze certain types of polysaccharides, reducing bacterial virulence and rendering the pathogenic bacteria sensitive to host defenses, such as phagocytosis. They are currently used for disruption of the biofilm matrix, also playing an important role as adjuvants, to boost the host immune system, and as immunogens, for the production glycoconjugate vaccines [14].

Integrases and recombinases: Integrases are viral enzymes that allow lysogenic phages to integrate their genetic information into the host genome, while recombinases are essential for bacteriophage DNA replication. The latter enzymes currently constitute valuable tools for precise genome editing; these proteins have multitude of applications in the field of Synthetic Biology (SynBio), as they can generate a variety of gene modifications, including DNA deletions and insertions. These enzymes were originally exploited for genomic integration of synthetic circuitry, as well as to rearrange DNA segments; this was followed by their use in combinatorial and reversible DNA assembly methods, logic gates, analogue-to-digital converters, memory devices, and multiplexed DNA editing via recombination. Their current use has been expanded to include non-model microorganisms, that although may have the right phenotype for industrial use, are difficult to genetically engineer [15]. Recombination techniques that use phage proteins for genetic engineering include the Red system, that comprises three proteins from lambda phage, and RecET, that only requires two polypeptides, RecE and RecT, from the Rac prophage [16]. Many systems can carry out specific gene editing, through the design of either synthetic oligonucleotides or DNA cassettes that are homologous to the regions flanking the gene of interest. Recombineering and multiplex automated genome engineering (MAGE) tools have radically improved genome engineering capabilities. These methods provide powerful mechanisms to ease the genetic manipulation of model and non-model organisms [17].

The most common phage integrases used in synthetic biology are large serine integrases, originating from phages TP901-1, phiC31 and Bxb1, used to engineer robust sensors. These synthetic devices have been applied to mammalian systems, with the digital response simplifying the procedure and making it more amenable to small scale experiments [18]. More recent developments in this field include the recombinase-mediated cassette exchange (RMCE) system (2010) and the dual integrase cassette exchange (DICE) method, described in 2017 [19]; these two approaches are applied in the construction of memory genetic logic gates for detecting biological events [20].

The discovery of novel integrases and recombinases from additional prophages will increase the repertoire of available recombinase-based tools, as well as allow the design of further, more advanced, methods, including the construction of complex devices for SynBio applications [15].

Endolysins and Holins: Endolysins and holins are part of the protein arsenal deployed by double-stranded DNA (dsDNA) phages to release their viral progeny from bacteria [21,22]. Holins are small proteins that form pores (holes, hence their name) in the plasma membrane, causing cell lysis, while endolysins are peptidoglycan hydrolases that use the pores created by the holins to reach the bacterial cell wall and degrade the peptidoglycan constituent [23]. The bacteriolytic activity displayed by dsDNA phages, supports their potential use as antimicrobial agents against Gram-positive bacteria. Moreover, the high specificity displayed by bacteriophages, together with the lack of known human toxicity, and the fact that bacteria rarely develop resistance, validate the use of phages in a variety of useful applications such as therapeutic agents, food safety, prevention of foodborne diseases, and as disinfectants to sanitize work surfaces and equipment [24]. Although the action of endolysin is very effective against Gram-positive bacteria, Gram-negative organisms are more resistant to this enzyme, as they contain an additional external membrane, that protects the peptidoglycan layer from degradation. Endolysins contain two distinct areas, an enzymatic catalytic domain (ECD) and a cell wall-binding domain (CBD), which are connected by a linker. Chimeric endolysins, designed by molecular engineering, have favorably improved the properties of these enzymes by combining different protein domains and specifically targeting particular Gram-positive bacteria. The biotech company Micreos has commercialized one of these chimeric enzymes, an engineered phage endolysin that is active against both methicillin-sensitive and methicillin-resistant *Staphylococcus aureus* skin infections [11].

The high specificity displayed by endolysins in the degradation of different types of peptidoglycans has paved the way for the use of these enzymes as biosensors because these proteins provide a faster and more effective bacterial detection, as compared to antibody reactions. The production of soluble antibodies remains an expensive and time-consuming procedure, therefore, several new approaches rely on the use of engineered cell wall binding domains, modified from those present in phage endolysins, as recognition elements. These domains have produced better outcomes than the antibody-based approach, with the addition of not displaying any significant cross-reactivity [25,26]. The cell wall-binding domain of these polypeptides has also been successfully exploited for commercial uses, such as conjugation of the recognition element encompassed within the CBD with colloidal gold nanoparticles, an application currently playing an important role in the food industry [25]. On the other hand, holins potentially have a much wider range of action than endolysins, because if these proteins reach the bacterial plasma membrane, they cause cell lysis in both Gram-positive and Gram-negative bacteria [27]. In addition, holins can be engineered to contain an additional peptide that specifically binds, and targets, particular bacteria [9]. These characteristics confer holins the potential to be used in a wide variety of applications, including biotechnological bacterial control and gene therapy; in addition, they can be designed as cytotoxic proteins to destroy cancerous cells, or to generate highly immunogenic bacterial ghosts to manufacture vaccines [28,29].

Structural Murein Hydrolases: Virion-associated peptidoglycan hydrolases (VAPGHs) are used by phages to infect both Gram-positive and Gram-negative bacteria. VAPGHs are structural enzymes that share some functional features with bacterial endolysins. They are essential for phages to inject their genome into the host cells, as these proteins partially and locally degrade the bacterial cell wall peptidoglycan. A particular feature of VAPGHs is their high thermal stability, which facilitates the putative use of these enzymes in food technology. Interestingly, genetically engineered chimeric VAPGH enzymes, either alone or in combination with endolysins, display enhanced lytic activity both in vitro [30] and in situ [31]. The VAPGHs have been proposed as important candidates for the treatment of human and animal infections caused by *Staphylococcus aureus*, with essential applications in prophylaxis, as this microorganism is found on human skin [32,33].

Anti-CRISPR Proteins: These proteins include Clustered Regularly Interspaced Short Palindromic Repeats (CRISPR) and CRISPR-associated (Cas) systems (CRISPR-Cas), that constitute part of the arsenal bacteria can deploy to avert phage infection, thus representing a prokaryotic version of the adaptive immunity present in vertebrates. Although most of the Cas nucleases currently used are specific for DNA, some of these proteins can target RNA, such as type III (Csm/Cmr), type VI (Cas13) and type II (Cas9) [34], but only those nucleic acids that have been previously captured and incorporated in the CRISPR repeats. Phages can produce proteins that block the action of bacterial CRISPR-Cas systems, by either directly targeting the bacterial Cas proteins or by attacking the variant SpyCas9 protein, the polypeptide used by bacteria for genome editing. The recent discovery of these proteins will undoubtedly open a novel path in the fight against harmful bacteria [35].

The anti-CRISPR proteins are currently used in biotechnological applications, playing an important role in a variety of processes. There is also a collection of newly discovered phage proteins that increase the arsenal that could be called upon in the fight against pathogenic bacteria. These novel polypeptides include tail fiber proteins, capsid protein polymerases and exonucleases. Their biotechnological potential is already being exploited in a variety of fields, with uses including therapy, bacteria typing and detection, surface disinfection, food decontamination, drug delivery, and vaccine development. A definitive advantage is that phage protein manipulations and regulation are easily social accepted, and these engineered polypeptides can play a major role in scientific discoveries [36].

Currently, an array of additional applications is being developed for these polypeptides, including uses for phage protein characterization and as new genetic and molecular tools, because there still are large amounts of raw data that need to be analyzed because of massive DNA sequencing programs and functional analyses [37]. Moreover, phage enzymes, including hydrolases, play a crucial role in the destruction of bacterial cells, and the discovery of these proteins paves the way for the development of novel antibacterial drugs. More than 70% of the phage open reading fames (ORFs) do not correspond to genes currently identified and characterized in the GenBank data base, which complicates their analysis, making it much more time consuming. Fortunately, recently developed computational methods provide a more suitable interface to determine the properties of phage proteins, as well as enzymatic activity predictions. These novel approaches include the additional hallmark of converting protein sequences into digital features, a process that permits the identification of hydrolases and, consequently, establishes learning-based predictive models. This is facilitated by the recent creation of free servers, such as PVPredn, that can identify phage virion proteins from nucleotide sequences [38]; further assistance is provided by databases, such as IMG/VR v3 (created in 2016) that, although not specific for bacteriophages, represents the largest collection of viral sequences so far compiled [39]. Additional, more specialized, bioinformatic tools include efam, established by Zayed et al. in 2021, and described by the authors as “an expanded collection of Hidden Markov Model (HMM) profiles that represent viral protein families conservatively identified from the Global Ocean Virome 2.0 dataset” [40].

## 3. Phage Based Methods

**Figure 1:**
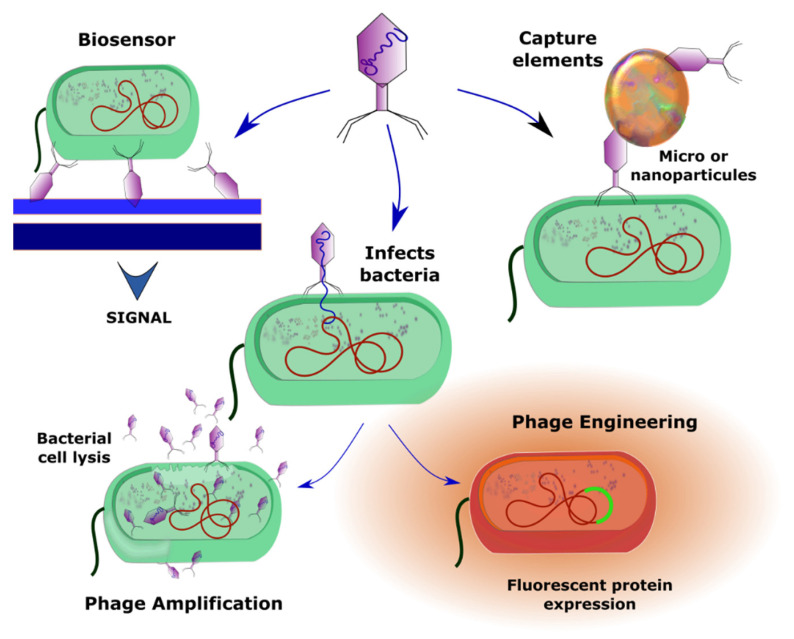
Bacteriophage-based methods for specific bacterial detection. When phages infect cells and multiply using their molecular machinery, their progeny can only be released after cell lysis. Genetically modified phages can infect and facilitate bacterial detection. The figure depicts a phage containing a gene that produces fluorescence and, when inserted into the bacterial genome, using a recombinant tempered phage, it facilitates bacterial detection. Reflected here are also phage uses as capture elements and as bioreceptors in biosensors. Modified from Richter et al. (2018) [41].

New techniques have also been recently developed in this area of research by taking advantage of phage capabilities such as protein–protein interactions for the development of novel diagnostic assays using bacteriophages to identify a variety of specific compounds (Figure 1).

Conventional culture-based methods and molecular detection mechanism remain as the standard procedures used for pathogenic bacteria determination, despite that these methods are slow and laborious [42,43]. This approach results in an estimated 30–50% of the patients receiving ineffective antibiotic therapy, with the additional drawback of antibiotic misuse known to contribute to the global spread of antimicrobial-resistant bacteria. Both, complete phages and their encoded proteins have been used in the development of a variety of diagnostic assays for bacterial detection. The inherent phage characteristic specificity in host infection makes these organisms ideal candidates as bio-probes for bacterial identification, in order to detect low levels of viable bacteria present in either food, water or clinical samples [44,45]. According to the ISO 11290-1:2017 guidelines for *Listeria monocytogenes* detection, identification of the bacteria requires a minimum of 48 to 120 h; the ISO guidelines recommend a minimum of 24 h for bacterial colony formation, on solid culture media, for fast-growing bacteria, plus an additional 24 h (96 h for slow-growing bacteria), to enable complete morphological identification of *L. monocytogenes* colonies [46]. On the other hand, newer bacterial detection and identification methods, such as nucleic acid amplification, ELISA-based antigen detection, matrix-assisted laser desorption/ionization time-of-flight mass spectrometry (MALDI-TOF-MS) and whole-genome sequencing (WGS), are much more labor and time efficient, although nucleic acid-based methods have the disadvantage of not being able to differentiate between DNA from either viable or dead cells. Moreover, the use of WGS and MALDI-TOF-MS poses a significant challenge for the food industry, due to the low numbers and the variability of the bacteria present in product heterogeneous matrices [47]. The implementation of robust and sensitive methods, such as phage-based diagnostics provide a viable alternative to eliminate these inherent problems [48].

Phages have evolved highly efficient ways to attach to bacteria, in a highly specific manner through mechanisms involving phage receptor binding proteins (RBPs). The use of bacteriophages for biotechnological assays provides specific advantages, such as the resilience these organisms display in harsh environments, and the fact that they can discriminate between dead and live bacteria. Moreover, phages can detect very small bacterial amounts, as by replicating inside their host cells they greatly amplify the detection signal in each viral reproductive cycle [49]. Phage-based sensing methods also have the advantage of being inexpensive and robust, maintaining a stability under unfavorable conditions, such as high temperatures or in the presence of solvents, that is far greater than that displayed by antibodies [50].

Bacteriophage typing has been one the most common techniques used to identify bacteria present in complex sample matrices [51]. This process can even discriminate between bacterial strains by using either using the lytic activity of whole phage particles, or just the phage proteins that confer host-binding specificity, such as cell wall-binding domains (CBDs), RBPs and phage endolysins. Hence, it comes as no surprise that these proteins have been successfully used in the detection of several Gram-positive bacteria, such as *Listeria* [52], *Bacillus cereus* [53] and *Clostridium tyrobutyricum* [54]. In addition, *Listeria*-targeting CBDs have been demonstrated to recognize a variety of *Listeria* serovars [55], while *Clostridium tyrobutyricum*-targeting CBDs have been reported to identify even spores involved in cheese spoilage. Moreover, clinical detection of pathogenic species, such as *Mycobacterium tuberculosis*, *Yersinia pestis*, *Bacillus anthracis*, and *S. aureus*, can be achieved by using bacteriophage-based methods. Buth and colleagues [56] deciphered the mechanism of interaction between RBPs and *Pseudomonas aeruginosa*, using R-type pyocins as RBPs models; R-type pyocins are bacterial antimicrobial peptides that resemble the tail structure of phages. Sonja Kunstmann and colleagues [57] pioneered the application of phage RBPs for bacterial identification. By using fluorescently labelled tail spike proteins from bacteriophage Sf6, they developed successful probes for *Shigella* detection. Recently, using a combination of *Listeria*-specific CBDs and RBPs, Meile and coworkers developed a glycotyping approach to identify *Listeria* serovars [55]. The relevant domains of the proteins can be used to coat magnetic beads, and specifically capture *Listeria* target cells, thus increasing sensitivity and allowing fast diagnosis [52]. This approach can also be used to purify bacterial cells, as it reduces, or even eliminates, contaminants, and increases target bacteria recovery. Uchiyama and colleagues [58] developed a procedure that allowed the removal of *Enterococcus faecalis* from vaginal samples.

### 3.1. Phage Display

**Figure 2:**
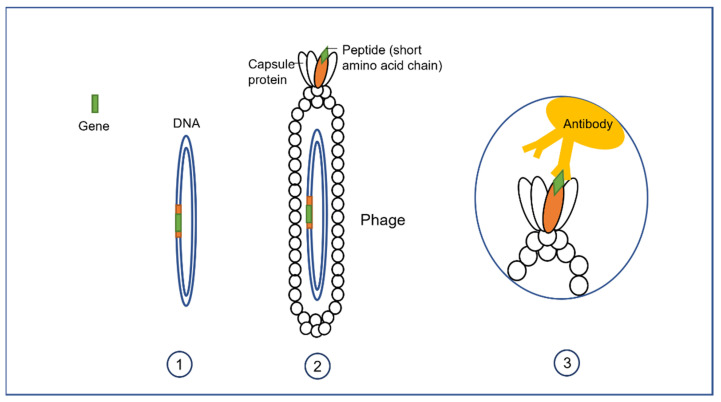
Summary of the technique designed by George Smith, during his time at the University of Missouri, which laid the foundations for the phage display technology. 1: A foreign gene is introduced into the coding region of a viral capsid protein, and expressed as a fusion with the phage polypeptide. The viral DNA is then inserted into bacteria, allowing the phage to multiply. 2: The peptide encoded by the heterologous gene, fused to a capsid protein, is displayed on the surface of the bacteriophage. 3: The final refinement devised by Smith was the use of an antibody, that recognized the foreign peptide, to identify the phage particles displaying the fusion protein. Currently, phage display constitutes a very powerful molecular biology technique, extensively used in many areas of research. This method plays a pivotal role in the identification of novel target ligands, a procedure that requires the construction of specific peptide libraries, that are expressed on the surface of the bacteriophage fused to a viral protein.

The protein–protein interactions that occur in phage particles are good examples of dipole-dipole interactions, such as hydrogen bonds, that constitute van der Waals molecular forces. As molecular studies demonstrated that only a region of the protein, denominated ‘epitope’, is responsible for antibody-antigen interactions, research has concentrated on identifying novel peptides that display high affinity to specific target cells. These bacteriophage studies culminated in the technique known as “phage display”, which allows the construction of peptide libraries fused to a phage protein and expressed on the surface of the bacteriophage, that are screened to identify novel target ligands [59]. George Smith, during his research at the University of Missouri, developed the phage display technology (Figure 2), an achievement for which he was awarded a Nobel Prize in chemistry in 2018 (shared with Greg Winter and Frances Arnold; [1]). Phage display technology, due to its intrinsic ability to display foreign antigens, can be used for a wide variety of purposes, ranging from the identification of phage elements suitable to use in vaccines against infectious disease and immune therapy, to cancer applications [60,61,62,63]. This technique can also be used to identify the autoantibodies responsible for some autoimmune diseases, such as a brain-specific E3 ubiquitin ligase, implicated in neurodegenerative disease processes, and TGIF2LX, generated in non-small cell lung cancer (NSCLC) [64].

Filamentous phages, belonging to the Inoviridae family, are typically around 900 nm long and 7 nm wide [65]. This family includes bacteriophage M13, which is widely used in phage display protocols. An advantage of using filamentous organisms in phage display systems is that all five coat proteins that integrate the virion can be used to display antigens. Moreover, filamentous phages can multiply inside the host and be released without killing the bacterial cells, which allows phage production in a sustainable manner. The release of their progeny from the host without contamination by bacterial lysates makes this method suitable for the rapid development of vaccines [1].

Bacteriophages can be genetically engineered to carry foreign peptides fused to their capsid proteins, hence, producing multitude of viral particles that display the recombinant peptides on their surface. Knowledge of both the sequence of the peptide used in the display and the proteins it binds to allows to establish a direct linkage between the genotype and phenotype of the proteins of interest. Compared to other cloning techniques, phage enrichment rapidly increases the number of copies of the peptides which, in turn, considerably expands the sensitivity of the procedure, thus boosting the chances of success in identifying currently unknown bait-binding peptides. Phage display systems can be classified, according to the bacteriophages used, into lytic and non-lytic (lysogenic). As mentioned above display vectors constructed from filamentous phages, such as M13, are non-lytic. A characteristic of filamentous phages is that they contain five capsid proteins (pVIII, pVI, pVII, and pIX), and the library of proteins to be analyzed can be fused to the N-terminus of any, or all, of them [66]. Techniques involving these bacteriophages are used for the discovery of specific diagnostic biomarkers as well as for the identification of new mimotopes (molecules that mimic the epitope structure), with applications in both the therapy and prophylaxis of a variety of diseases, including tuberculosis [67]. New advances, in both phage display technology and in the structural knowledge of bacteriophages have led to the development of a novel type of phage display libraries, known as “landscape phage”, involving the display of nanomaterials on the phage surface [68]. This novel technique considerably expands the applications of phages into a variety of different areas, such as bioscience, medicine, material science, and engineering [69].

In fact, there are currently even commercially available phage display systems which are the basis for most of the studies carried out at present. Three of the available systems, Ph.D.-7, Ph.D.-12 and Ph.D.-C7C, offer the possibility of testing 12 linear amino acid residues, 7 linear residues, and either cyclic (via cys-cys disulfide bond) or 7 random peptides, respectively. Due to the current demands in this area of research, it is predictable that new improvements and additional techniques will become available in the near future [50].

Phage display has not been used for the study of protein-protein interactions in functional proteomics, and techniques such as the yeast two-hybrid system, protein affinity purification, tandem affinity purification coupled with 1D or 2D gel electrophoresis, and mass spectrometry (AP-MS or TAP-MS), remain essential in the field. However, recent improvements and modifications in the procedures involving phages, such as C- terminal display and ORF cDNA libraries, are starting to outline a role for bacteriophages in that field. In fact, recent successes, such as the use of an ORF phage display to efficiently identify tubby-N-binding (tubby proteins are cell signaling proteins present in eukaryotes) and PS-binding proteins (PS stands for phosphatidylserine) [70,71] demonstrate that ORF phage display is an efficient, sensitive, and versatile technology for the elucidation of specific protein-protein interactions involved in either disease mechanisms or as possible therapeutic targets. Moreover, these findings indicate that ORF phage display has the potential to join, or even displace, the yeast two-hybrid system and AP/TAP-MS as one the main techniques in functional proteomics [72].

Recently, a novel approach described the use of phage display in combination with antibody technology and MS, which was successfully used for the identification of cell- type specific protein markers. Phage display, in conjunction with MS, can detect, identify, and analyze both secreted and membrane-associated extracellular proteins as well as different cellular structures, as demonstrated by Jensen and colleagues for the identification of keratinocyte-specific markers [73].

Table 1 is a selective summary of currently available therapeutic agents that are derived from phage display technology [59].

### 3.2. Phage Assisted Evolution

**Figure 3:**
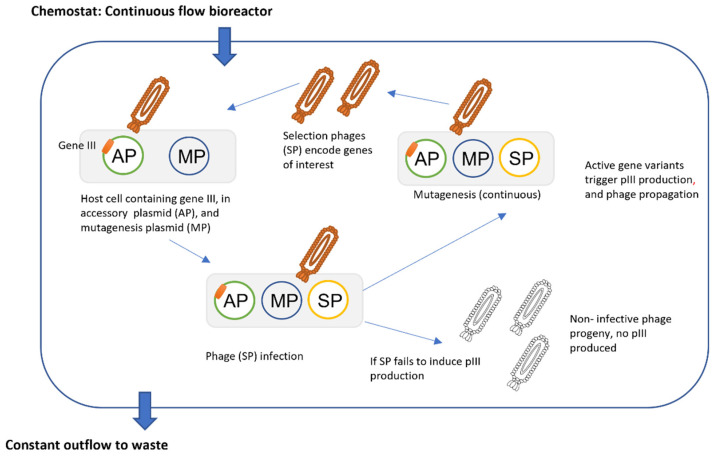
Overview of PACE (Phage-Assisted Continuous Evolution) in a single vessel. This technique allows continuous, rapid, protein mutagenesis and selection, under specific pressure designed for the experiment. Bacterial host cells continuously flow through a bioreactor, where they are infected with a population of replicating phage DNA vectors. The bacteriophages used in PACE lack gene III, encoding protein III (pIII), which is essential for bacteriophage infection; this gene is provided in AP, the accessory plasmid. Selection phages (SP) code for genes of interest; they are part of a plasmid library, that contains DNA fragments. Only the functional members of the plasmid library induce production of pIII, from AP, and release progeny capable of infecting new host cells. Increased mutagenesis is triggered by the mutagenesis plasmid (MP), that can be induced to produce mutagenesis genes. Host cells flow out of the bioreactor, on average, faster than they can replicate, hence confining the accumulation of mutations to the replicating phage.

Phage-assisted continuous evolution (PACE) is a technique, reported in 2011, that allows continuous, rapid, protein mutagenesis and selection, under specific pressure designed for the experiment [89]. This is a phage-based technology that makes it possible to carry out directed evolution of proteins in bacteria, without the time scale required for classic Darwinian evolution. The procedure requires a continuous system, with evolving genes transferred from one host bacteria to another, by means of a modified bacteriophage cycle. The phages used in PACE lack gene III, encoding protein III (pIII), which is essential for bacteriophage infection; this gene is provided in AP, the accessory plasmid. Mutagenesis is triggered by the mutagenesis plasmid (MP), that can be induced to produce mutagenesis genes. Selection phages (SP) code for genes of interest; they are part of a plasmid library, that contains DNA fragments. Only the functional members of the plasmid library (those that produce the activity for which the experiment has been designed) induce production of pIII, from AP, and release progeny capable of infecting new host cells; hence placing the bacteriophages under continual evolutionary pressure. This evolutionary pressure is what hastens the rate of mutation, resulting in dozens of evolution rounds occurring in a single day (Figure 3). The PACE technique has many advantages, including the fact that its execution requires a minimal research effort. Phage-assisted non-continuous evolution (PANCE) is a similar method that follows the same principles as PACE; the main difference between the two procedures is that PANCE requires serial dilutions, instead of continuous flow. The PANCE method also permits multiplexing (evaluating multiple targets in a single experiment) phage evolution, providing substantially more information, per assay, than PACE; the only drawback is that this technique is slower than PACE [90].

A more recent development, phage- and robotics-assisted near-continuous evolution (PRANCE), is an automated system that carries out phage-assisted continuous evolution in high-throughput, allowing up to 96 experiments to be concurrently performed. An additional advantage of PRANCE is that it uses real-time monitoring of biological activity, and can adjust selection stringency, through an automated feedback control system [91].

DeBenedictis and coworkers [91] demonstrated the real-time monitoring capability of this system in 2022. The authors engineered an M13 bacteriophage encoding T7 RNA polymerase (RNAP) but lacking the pIII phage coat protein; the bacterial host strain expressed pIII together with a luminescence reporter (luxAB), both genes were under the control of a T7 promoter. This allowed real-time monitoring of the engineered phage propagation, by detection and quantitation of the luminescence produced [91].

### 3.3. Phage Amplification-Based Detection

The phage amplification assay starts with the bacteriophage being cultured in suitable bacterial cells, as it is the viral progeny that is used for the assay; once collected, the newly released viruses are inactivated and washed. Unfortunately, the requirement for a phage amplification step has some disadvantages, particularly in the case of prophages that integrate into the bacterial genome (lysogenic cycle). For prophages to undergo the normal process of viral reproduction and release of phage progeny they must switch from the lysogenic to the lytic cycle, which can allow the host cells to unleash molecular mechanisms that alter, or even stop, the phage productive cycle [8]. Despite these drawbacks, this method has been successfully applied in the detection of bacteria, such as *Listeria* spp. and *Mycobacterium* spp., in milk.

Amplification also considerably increases the number of viral particles, but this is a minor problem as the number of bacteriophages, as well as the phage titer, can be easily determined by procedures that involve either techniques such as ELISA (enzyme-linked immunosorbent assay) or by directly measuring the phage nucleic acid content. The sensitivity of the phage amplification assay is often increased by capturing and enriching the phage particles, using either lateral flow assays or magnetic beads. Additional methods involve procedures that use reverse transcription to make cDNA from the phage RNA, and this methodology was demonstrated to be more sensitivity than DNA amplification-based detection systems. Furthermore, some procedures take advantage of the phage lytic cycle to detect viable bacterial cells. Because only living cells are infected by bacteriophages, when lysed they release their contents, including ATP, which can be easily detected using bioluminescence-based reactions. Similar methods have demonstrated a change in redox reactions in *Salmonella enterica*, *S. typhi* and *S. paratyphi* after phage infection [92]. Luo and colleagues successfully used a combination of enrichment and phage-based qPCR assays to rapidly (in 6 h) detect *Acinetobacter baumannii* in sputum samples from patients with lung infections, using p53 phages [93]; similarly, it only took 10 h for Garrido-Maestu and coworkers to detect *Salmonella enteritidis* in chicken flesh [94,95]. Phage amplification procedures described in the literature include: (i) Detection of *E. coli* by a technique using antibody-conjugated beads to isolate amplified MS2 phages [96], and (ii) pathogenic *E. coli* and *Salmonella* Newport identification in food samples by using a procedure involving phage coated paper dipsticks and qPCR-mediated detection of viral progeny [97].

### 3.4. Phage Engineering

Recombinant phages can be engineered to produce a detectable signal when replicating in bacterial cells, and this signal can function as an indicator of cell viability [98]. A variety of genetically engineered phages incorporate genes encoding for either fluorescent markers such as luciferases, or hydrolyzing enzymes such as β-galactosidase, that are easily detected [8,48]. Although these applications have been successfully used, the fact remains that genetically modified bacteriophages are often less infectious than the wild-type viruses. In addition, the environmental risks that would involve an inappropriate release of these organisms into nature need to be taken into consideration. Recent reviews describe the practical applications of modified phages in phage therapy, medicine, animal industry, and agriculture, as well as for use as antimicrobials, biocontrol agents and genetic engineering tools [99].

Some genetic engineering approaches that enhance the sensitivity of procedures for the detection of bacterial products that are released after phage infection and cell lysis use strong recombinant promoters to overexpress the relevant proteins within the bacterial host, thus producing a strong signal that is easy to identify [100]. To achieve this, the phage must infect the bacteria and introduce the reporter gene into the target pathogen (e.g., *Salmonella*, *Campylobacter* and *E. coli*) [101]. This also requires the identification of a suitable region in the phage genome that would allow integration of the reporter gene without disrupting infectivity [48].

An alternative approach to detect the presence of viable host cells requires the phage to carry a reporter gene that may be detected through enzymatic substrate conversion. Phages can be genetically engineered using three different procedures, direct cloning, homologous recombination with or without CRISPR-Cas counter selection, and whole genome activation.

Direct cloning involves the use of phage vectors, plasmids or phagemids containing an additional origin of replication and a packaging sequence from a phage. However, this procedure can only be used in mycobacteriophages and some phages of Gram-negative bacteria, with the additional disadvantage that their packaging capacity is relatively small [102,103]. The method that uses CRISPR-Cas systems coupled to homologous recombination facilitates the enrichment of recombinant phages [104]. A *Listeria ivanovii* type II-A CRISPR-Cas system was successful in modifying the lytic *Listeria* phage A511, generating two variants that introduced bioluminescence genes into *Listeria* spp. [105].

Luciferase is the main reporter protein used in these applications. One of these methods involved the introduction of the *Vibrio harveyi* luciferase (luxAB) gene into the genome of phages, to create recombinant bacteriophages that infect bacteria and use the host cell machinery to produce bioluminescence. In the first published work, a phage encoding NLuc was inserted into *E. coli* phage ΦV10, with the aim of detecting *E. coli* O157:H7 [106]. Additional luciferase-based constructs designed to identify *L. monocytogenes* live cells contained luciferase coding sequences from *Vibrio harveyi* (luxAB), *Gaussia princeps* (gluc), *Renilla reniformis* (rluc) or *Oplophorus gracilirostris* (oluc) inserted into the *Listeria* phage A500. Further progress included the design of nluc-containing Myovirus A511 (A511::nlucCPS) in a system that can detect a single *L. monocytogenes* cell present in food samples [107]. Additional developments include the design of T7-based phages, encoding an NLuc-carbohydrate-binding module fusion protein (NLuc-CBM) for the identification of *E. coli* contamination in both water and food samples [108,109]. The sensitivity of the assays was improved (to detect 1 CFU/100 mL) by using cellulose-coated beads to concentrate and purify NLuc-CBM [110]. The T7 phages encoding alkaline phosphatase were successfully used to detect *E. coli* using substrates such as p-nitrophenyl phosphate (pNPP), that is hydrolyzed to p-nitrophenol (pNP) [111], nitro-blue tetrazolium chloride NBT and 5-bromo-4-chloro-30-indolyphosphate p-toluidine salt (BCIP) [112,113]. Moreover, another fluorescence application reported the use of a T7-ALP phage to detect *E. coli* in beverages [114].

The gene encoding green fluorescent protein (GFP) was introduced into HK620 and P22 phages to detect *E. coli* and *Salmonella enterica* Typhimurium contamination, respectively. Detection was performed by flow cytometry, and the limit of detection (LOD) observed was 10 cells/mL of seawater. The same group [101] also reported the engineering of two phages (HK620 and HK97), containing an entire luxCDABE operon incorporated into COMBITOX, that achieved an LOD of 10^4^ bacteria/mL. The COMBITOX is a multi-parameter instrument that measures toxins. It contains several biodetector systems that permit detection of a variety of pollutants, including bacteria, toxins, and heavy metals [101]. Rondón and coworkers [115] used the *mCherry_bomb_*φ phage for the detection of *Mycobacterium* spp. in patients suffering from tuberculosis, as well as for phenotypic determination of rifampicin resistance.

Receptor Binding Proteins (RBPs) are the polypeptides used by phages to target specific molecules on the bacterial wall; these RBPs are responsible for the specificity and limited host range of bacteriophages. The implication is that RBPs could be engineered to increase the host range of particular phages, an approach that would benefit areas of research such as the use of bacteriophages in human an animal therapy. Dunne and coworkers [116] used different approaches to achieve what they describe as “structure-guided receptor binding protein (RBP) engineering”; the methods described included the creation of chimeric RBPs, targeted mutagenesis and homologous recombination. These authors developed an R2 pyocin (pyocins are bacteriocins produced by certain *Pseudomonas aeruginosa* strains, that have a structure similar to a simple phage tail) as a platform to analyze the RBPs they engineered. In addition, Yehl et al. [117] identified the host-range-determining regions (HRDRs) in the tail fiber protein of the T3 phage and used an approach similar to antibody specificity engineering to generate diversity in them and, hence, expand their host range. The authors used a high-throughput targeted mutagenesis technique to create changes, on the regions of HRDRs identified as crucial for host recognition. This procedure generated a great variety of ‘phagebodies’ (as the authors denominated the mutated constructs), as many as 10^7^ synthetic variants according to the researchers, that still maintained the structural integrity of the phage tail, while displaying different host specificities. The phagebodies were tested, on a mouse skin infection model, by their ability to kill bacteria. This technique will be invaluable, as it can expand the host range of phages, in the design of novel bacteriophages as therapy agents to combat human and animal infections [104].

### 3.5. Biosensors

**Figure 4:**
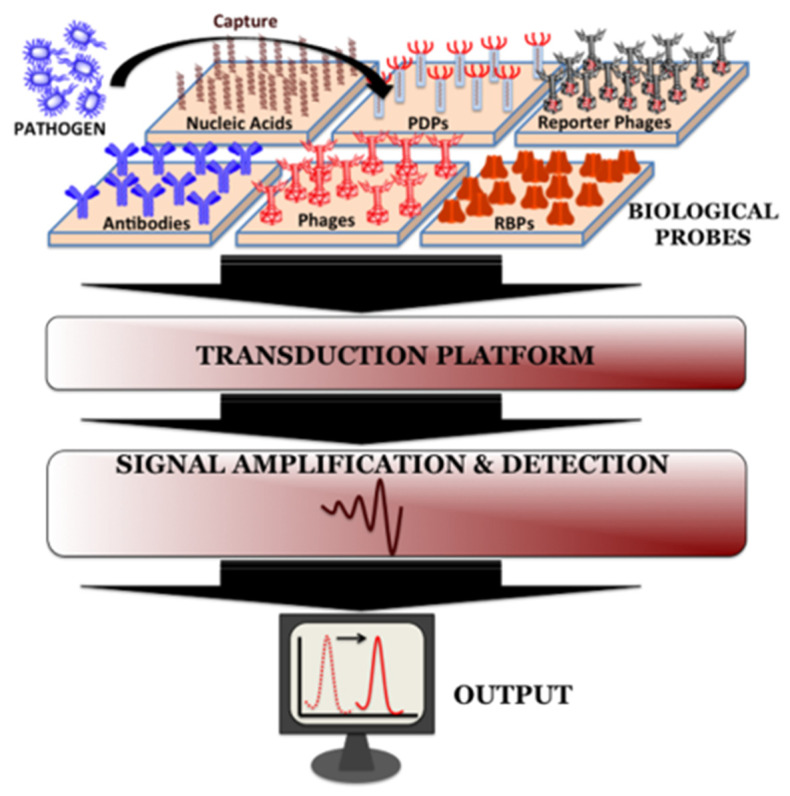
Components of a typical biosensor for pathogen detection, highlighting the currently available phage-based molecular probes. A standard biosensor contains three associated components: (i) a sensor platform, displaying bioprobes that confer specificity of recognition, (ii) a transduction platform, that generates a measurable signal when the bioprobes successfully capture target molecules, (iii) and the amplifier, which enhances and processes the signal in order to provide a quantitative estimate of the target molecules captured. Figure from Singh et al., 2019 [111] (Creative Commons Attribution License).

Critical parameters that define the applicability of sensors include limit of detection, time of analysis, sensitivity, and specificity [8]. The use of bacteriophages in combination with a variety of transducers has led to the development of new biosensors, with advanced bioanalytical capabilities enabling the identification of novel biomarkers. The design of a peptide-based biosensor requires two main stages: (i) receptor selection, and (ii) functionalization by selecting the appropriate synthetic peptides [118,119,120,121]. Biosensors are commonly used in a variety of roles, that include the identification of bacteria, detection of complete phage particles, and recognition of molecules such as peptides (Figure 4). Recently, RBPs have been used as novel sensing elements that provide binding capabilities equivalent to those displayed by whole phages, but being considerably smaller, they facilitate the construction of highly effective diagnostic tools [48,49,122,123]. The RBPs can also be used in ELISA-based assays [57,124] as well as for glycotyping *Salmonella* [10] and *Listeria* [125]. In addition, cell binding domains (CBDs) of phage endolysins can be used in biosensors to identify Gram-positive pathogens, such as *B. cereus* [53] as well as in lateral flow assays [25] and procedures involving magnetic enrichment-based detection [126].

Sensors can be classified into different groups, depending on whether they use electrochemical (voltammetric, potentiometric, impedimetric) or optical (fluorescent, surface plasmon resonance, surface-enhanced Raman spectroscopy) transducers. Bacteriophages can be attached to solid substrates, using three main procedures: (i) electrochemical methods, where phages are deposited on the electrodes, (ii) magnetoelastic sensors, where a change in mass changes the amplitude of vibrations, and (iii) by surface-enhanced Raman spectroscopy, where excited plasmons within the substrate produce an increase in spectral intensity.

Electrochemistry-Based Detection: Electrochemical biosensors are sensitive and specific, as well as requiring a low-cost, simple process. Several recent publications review the development of phage-based electrochemical methods, either for the detection of bacteria [127,128,129] or for disease diagnosis [130].

When recombinant enzymes are introduced into the bacterial targets, their enzymatic activity can be detected and monitored by measuring the levels of the products released from their substrate; for example, β-galactosidase hydrolyzes 4-aminophenyl-β-d-galactopyranoside (PAPG) and produces 4-aminophenol (PAP). The electroactive PAP product is easily quantitated by amperometry, a method that uses electrical currents to detect ions in solution [110]. To increase the sensitivity of electrochemical biosensors, biomarkers can be immobilized on the surface of electrodes. One of these improved techniques involves the use of engineered T7-based phages with a gold-binding peptide fused to an alkaline phosphatase enzyme (GBPs-ALP) that, upon release during bacterial cell lysis, bind to the surface of the gold biosensor, where they are detected [110]. The activity of GBPs-ALP-coated electrodes was then electrochemically quantified using linear sweep voltammetry (LSV). This method allowed the authors to detect, in drinking water, phage colony counts as low as 10^5^ CFU/mL, in just 2 h [48,110]. Meile and colleagues also described how a single phage can detect multiple *E. coli* strains [48]. Yue et al. [131] analyzed the detection of *Pseudomonas aeruginosa* by a label-free biosensor, using electrochemiluminescence, reporting a LOD of 56 CFU/mL within 30 min. The design of magnetoelastic sensors allowed the detection of methicillin-resistant *Staphylococcus aureus*, with a limit of 3 × 10^3^ CFU/mL, within 30 min [132]; these sensors could also detect *Salmonella* as a surface contaminant in food products (*S. enterica* in chicken and *S. typhimurium* in lettuce). Moreover, the advent of surface-enhanced Raman spectroscopy (SERS) provided a boost in the intensity of recorded spectra. This advancement led to the development of novel, improved, commercially available substrates, such as *SERSitive*, that are increasing the range of detection by these techniques, as in the case of an application that uses thin silver films and T4 phage on a silicon platform, which increased its LOD from 10^7^ to 10^8^ [8]. Other improvements resulted in an *E. coli* LOD of 1.5 × 10^2^ CFU/mL, and the successful use of *Tbilisi* bacteriophages in a SERS-based system designed to detect *Brucella*. In addition, Paczesny and coworkers used gamma phages for the detection of *Bacillus* species [8].

Phage-based sensors are also useful in disease diagnosis. Prostate-specific membrane antigen (PSMA) is a biomarker of prostate cancer that can be detected in either urine or semen. Yang et al. described in 2006 [133] one of the first virus-based sensors to diagnose prostate cancer. These authors designed M13 bacteriophages displaying a PSMA-binding sequence on the N-terminus of pVIII coat protein (immobilized on the surface of a gold electrode via an N-hydroxysuccinimide thioctic ester (NHS-TE) linker) that was monitored by quartz crystal microbalance (QCM) and electrochemical impedance spectroscopy (IES) [133]. This approach was also used to develop sensors for the detection of a Dengue virus type 2 marker (DENV2 NS1 protein), as well as troponin I, myoglobin, alanine aminotransferase, and a *Norovirus* coat protein [118,119,120,121].

The term ‘virus bioresistor’ (VBR) refers to a device that contains virus particles (e.g., M13 bacteriophages) directly connected to an electric circuit, usually linked to PEDOT (poly3,4-ethylenedioxythiophene) [134]. A virus-PEDOT impedimetric sensor was investigated as a tool to detect the presence of human serum albumin (HSA) in the urine of patients suffering from kidney or bladder diseases [130]. This system was later modified to detect the DJ-1 protein, a multifunctional human protein involved in immune and inflammatory diseases [135].

Other electrochemistry-based sensors, based on M13 Bacteriophage/Peptide Sensors, recently developed include Light Addressable Potentiometric Sensors (LAPS), which use variations in electric potential as a measurable chemical signal, Surface Plasmon Resonance and Surface-Enhanced Raman Spectroscopy [136,137].

Fluorescence-Based Detection: Most of the fluorescence-based reporter phage assays developed to date focus on either the detection of *Mycobacterium*, or on drug susceptibility testing (DST). Engineered phage-mediated fluorescence was designed to monitor drug-resistant bacteria, which can be detected by either fluorescence microscopy or flow cytometry [138,139]. The fluoromycobacteriophages engineered so far are mainly derived from the TM4 temperate phage [140,141].

A recently developed interesting technique involves the in vivo imaging of specific bacterial cells by M13 bacteriophages carrying affinity peptides and chemical labels, such as fluorescent dyes or nanoparticles, as well as highly selective labelling agents (to target the relevant bacteria). This method represents the first selective staining ever developed for bacteria [142].

Capture elements: One of the main problems in the identification of either molecules or bacteria is the need to detect very small amounts, as both the cells and the compounds can be in very low concentrations in any given sample. Some of the recent methods used to increase sensitivity involve either micro- or nano-particles conjugated with bacteriophages. This approach, which considerably increases surface (detection) area, has been used for the detection of bacteria [143,144]. The high binding affinity displayed by whole phage particles has undoubtedly led to their use as bio-probes in biosensors, either in conjugation with radioactive tracer fluorophores, magnetic nanoparticles, or both [145,146]. One such example involved the use of gold nanoparticles, together with phage P9b, to detect *Pseudomonas aeruginosa*, by surface-enhanced Raman spectroscopy (SERS) [147]. An additional study attached gold nanoparticles (AuNPs) via SH groups to the surface of genetically engineered M13 phages that displayed receptors against a variety of target bacteria (two strains of *E. coli*, *P. aeruginosa*, *Vibrio cholerae*, and two strains of the plant pathogen *Xanthomonas campestris*). This assay could detect 10^2^ cells (per mL of sample) in a 30-min procedure [148]. Bacteriophages can also be immobilized onto the surface of core-shell SiO_2_@AuNP nanoparticles, that contain silica cores that by causing aggregation, generate strong light scattering which allows detection. This technology was applied to identify *Staphylococcus aureus* contamination, with a LOD of 8 × 10^4^ CFU/mL, detected in only 15 min (Imai et al., 2019) [149]. The authors also used this technique in an application to diagnose the presence of *S. aureus* in apple juice for which a LOD of 9 × 10^3^ CFU/mL was achieved. Other molecules used for phage conjugation and bacterial detection include Fe_3_O_4_ particles (Liana et al., 2017) [150], used for the rapid capturing and isolation of *E. coli*, as well as RMOF-3 (Zn_4_O(NH_2_-BDC)_3_) (NH_2_-BDC = 2-aminoterephthalic acid), conjugated with isolated lytic bacteriophages, used as fluorescence probes. Additional examples include: (i) the detection of *Staphylococcus arlettae*, with a LOD nearing 10^2^ CFU/mL (Bhardwaj et al., 2016) [151]; (ii) the use of amine functionalized isoreticular metal-organic framework-3 (IRMOF-3) and another metal-organic compound (NH_2_-MIL-53(Fe)), to detect *S. aureus* with a LOD of 31 CFU/mL, in 20 min (Bhardwaj et al., 2017) [152]; and (iii) nanomaterials such as Cu_3_(PO_4_)_2_ nanoflowers, loaded with glucose oxidase and gold nanoparticles, attached to T4 phages, which were successfully used for bacteria detection, achieving a LOD of 1 CFU/mL within 140 min (Paczesny et al., 2020) [8].

## 4. Mass Spectrometry (MS)-Based Proteomics

**Figure 5:**
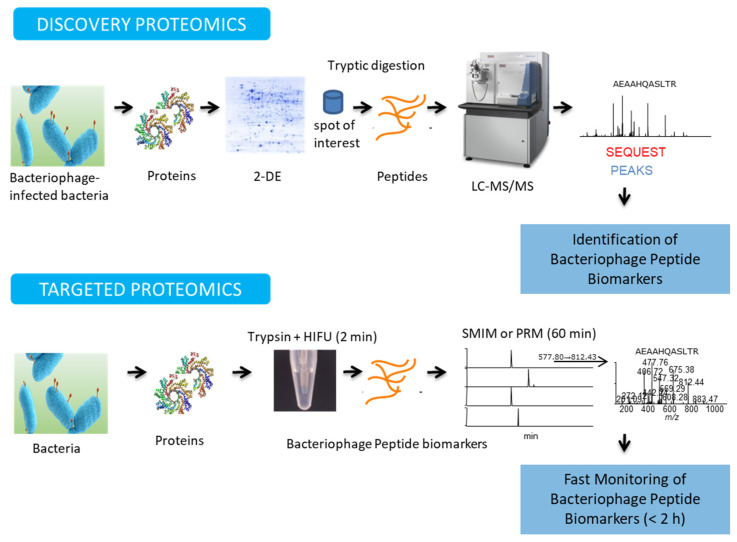
Sequential workflow required to engineer bacteriophage-based mass spectrometry proteomics (Discovery proteomics and Targeted proteomics). Discovery proteomics: protein extracts, from bacteriophage infected bacteria, are purified and separated by two-dimensional gel electrophoresis (2-DE) and stained. The proteins of interest, that appear as spots, are then excised from the gel, and digested with trypsin overnight; with the resulting peptides analyzed by liquid chromatography, coupled to tandem mass spectrometry (LC-MS/MS). The MS spectra obtained permits peptide identification, using search engines such as Se-quest; in the case that the MS spectra is not sufficient for peptide identification, the unknown peptides must be subjected to de novo sequencing, using platforms such as Peaks. The molecules identified as specific for a particular bacteriophage, are then selected as peptide biomarkers; these are the peptides to be monitored in targeted proteomics. Targeted proteomics: in this approach, the protein extracts are not first subjected to separation techniques, but directly digested with trypsin, using an accelerated approach that requires the use of high-intensity focused ultrasound (HIFU); this approach can reduce the protein digestion time to less than 2 min. The peptide biomarkers, selected in the discovery phase, are then monitored by mass spectrometry; a procedure that can be carried out by either selected ion MS/MS monitoring (SMIM) or parallel reaction monitoring (PRM). This targeted proteomics approach is very fast, requiring less than 2 h.

Figure 5 summarizes the common workflows involved in MS-based proteomics. It depicts the sequential steps required in two proteomics approaches, discovery proteomics and targeted proteomics.

Mass spectrometry-based (MS) methods offer several advantages, for bacteriophage identification, over other approaches such as sequencing-based methods; LC-MS-MS can precisely detect and identified bacteriophage peptides from an unknown sample, making this technique much faster, easier, and simpler than sequencing-based methods. The latter require purified molecules, a laborious and expensive procedure, as compared to MS. In addition, the MS approach does not require bacterial cultivation, as the samples for analysis can be directly collected from food. Furthermore, MS techniques can directly detect the viral proteins produced by prophages, while integrated in the bacterial genome, or by phages infecting the bacteria; these situations represent a serious challenge for DNA sequencing methods, as the genomic sequences of the phages are contaminated with the bacterial DNA [153].

### 4.1. Discovery Proteomics

Discovery/exploratory proteomics is used to analyze the proteome of a particular organism, to identify potential protein/peptide biomarkers. The most common method used to identify proteins is the so-called, bottom-up proteomics in which the proteins of interest are separated, thus reducing sample complexity, enzymatically digested, usually with trypsin, and the resulting peptides are analyzed by MS [154]. Bottom-up approaches can be classified into gel-based or gel-free methods, depending on how the protein separation step is carried out. Two-dimensional electrophoresis (2-DE), a gel-based strategy, is generally the method of choice to isolate individual proteins found in complex samples. In 2-DE, proteins are separated by their isoelectric point in a pH gradient in the first dimension, then gels are turned 90 degrees, and the proteins are separated by mass in the second direction. This procedure allows the separation of thousands of proteins in one single gel, and the resulting polypeptides can be visualized by in-gel staining [155]. As the isolated proteins appear as spots in the gel, they can be excised from the gel, digested by proteolytic enzymes, and the resulting peptides are then analyzed by MS for protein identification. This gel-based method is currently the prevalent option to analyze proteins in samples generated from organisms for which the nucleic acid sequence is either unknown, or only partially known. In these cases, protein identification is based on the comparison of the sequences of the peptides obtained by proteolytic degradation of the proteins excised from the gel with those of known orthologous proteins from related species, or by de novo MS sequencing [156]. In addition, current progress in this field, which improved the specificity and sensitivity of detection methods, makes 2-DE a good tool for the analysis of post-translational modifications (PTMs), such as glycosylation [157] and phosphorylation [158]. There are currently several software programs designed for 2-DE image analysis, such as PDQuest, Melanie and Progenesis [159]. In gel-free strategies, also known as shotgun proteomics [160], a complex mixture of proteins is directly enzymatically digested, and the peptides present in the reaction mixture are separated by reverse phase liquid chromatography (RP-LC), either alone or in combination with a multidimensional LC step, such as strong cation exchange chromatography (SCX), that uses a negatively charged ion exchange resin [161,162]. The individual peptides obtained are then fragmented and analyzed by tandem mass spectrometry (MS/MS) [163,164]. Using protein database search algorithms, like SEQUEST [165] or Mascot [166], fragmentation spectra are assigned to putative peptide sequences, and these assignments are then validated with programs like PeptideProphet [167] or Percolator [168]. If the protein/peptides are not present in the database, they must undergo de novo sequencing [169], a method that uses computational approaches to analyze and interpret the MS/MS spectrum obtained. These analyses can be carried out either manually or by using computer-assisted programs, such as Byonic [170] and PEAKS [171,172]. Protein quantification is commonly required in a variety of discovery/exploratory proteomic investigations. The main methods used in quantitative proteomics are: (i) isotope tagging by chemical reaction, such as isobaric tags that can achieve, either relative or absolute, quantitation (iTRAQ); (ii) tandem mass tag (TMT), that also requires different chemical labels; (iii) difference gel electrophoresis (DIGE), that involves labelling with fluorescent dyes [173,174,175]; ]; (iv) stable isotope incorporation, requiring an enzymatic reaction (i.e., 18O) [176]; (v) metabolic stable isotope labeling, such as stable isotope labeling with amino acids in cell culture, SILAC) [177]; and (vi) label-free quantification, a mass spectrometry method [178].

Top-down proteomics [179] is an alternative approach, that does not require protein digestion, with the intact proteins being directly loaded inside the mass spectrometer, where they are dissociated, and the resulting fragments analyzed. Novel dissociation mechanisms and MS improvements that provide increased resolution and better mass accuracy, resulted in the development of the new high-resolution MS (HRMS) instruments which are currently available [172,180]. The main goal of discovery/exploratory proteomics is to compare the resulting peptides and proteins with those in available data bases, using alignment search tools such as BLAST (https://blast.ncbi.nlm.nih.gov), to select particular peptide biomarkers [153] for use in targeted proteomics.

### 4.2. Targeted Proteomics

Targeted proteomics is a technique mainly used to monitor, with high sensitivity, accuracy and reproducibility, peptide biomarkers previously selected in the discovery/exploratory phase [181]. In this scanning mode, the MS analyzer is focused on evaluating the peptides of interest by either selected reaction monitoring (SRM) or multiple reaction monitoring (MRM) [182]. Monitoring appropriate pairs of precursor (parent ion) and fragment ions m/z (product ions), known as transitions, represents a sensitive MS technique to detect and identify peptide biomarkers. These are sensitive and selective methods, with a good signal-to-noise (S/N) ratio, an increased dynamic range and high reproducibility [183]. The SRM/MRM procedures are commonly performed on triple-quadrupole (QQQ) mass spectrometers; these instruments scan, either one or several peptide biomarkers, or the proteotypic peptides representing the protein of interest. However, the optimization of SRM/MRM assays is a time-consuming process and, more importantly, when in scanning mode, the complete MS/MS spectra is not registered; the MS/MS spectrum of a peptide is tremendously important to verify its amino acid sequence. New procedures in this field include SRM-triggered MS/MS in quadrupole-ion trap (Q-IT) mass spectrometers [182], selected MS/MS ion monitoring (SMIM) [184,185] and parallel reaction monitoring (PRM) in an ion trap (IT) or high-resolution Q-Orbitrap (Q-Exactive) instruments [186]. These techniques represent alternative scanning modes, with high sensitivity, for monitoring specific molecules and obtaining complete structural information. The high scanning speeds achieved by both the IT and the Orbitrap instruments, allow acquisition of the MS/MS spectra in a fraction of a second, recording the information obtained from the complete spectra, thus obtaining high-confidence MS/MS spectra, due to the option provided for averaging the signal during acquisition. Targeted data-independent analysis (DIA), implemented as sequential windowed acquisition of all theoretical fragment-ion spectra (SWATH-MS) [187], is an advanced MS mode that can identify and quantitate large sets of proteins, without having to specify a set of proteins prior to acquisition. Targeted proteomics strategies, in combination with stable-isotope dilution methods, such as 13C- or 15N- labeled absolute quantification peptide standards (AQUA) or concatenation of standard peptides (QCAT) [188], are labeling strategies introduced to the sample as internal standards, for absolute quantification of the proteins. Several bioinformatic software programs, such as Skyline [189] and SRMCollider [190] are currently available for the analysis of targeted proteomics assays.

The following sections will provide further information, and corroborate the importance of this operating mode, monitoring the peptide biomarkers identified and selected in the discovery/exploratory phase, for MS-based applications involving phage proteomic studies.

### 4.3. Identification of Bacteriophage-Derived Proteins for Bacteria Detection by MS-Based Phage Proteomics

A recent development involves the use of new MS techniques, such as MALDI-TOF MS and LC-MS/MS, for the identification of bacteria via the detection and identification of phage proteins (Table 2). These LC-MS/MS-based methods for bacteriophage identification offer many advantages over other approaches, because this methodology permits direct phage identification without a requirement for genetic tools. Bacteriophage detection and identification by a MS requires the production of phage progeny, a time-consuming process, but prophage detection can be carried out using protein biomarkers, as an alternative to genomic detection. Nevertheless, proteomic techniques allow the identification of several different bacteriophage species in a single analysis, which makes the procedure faster and cheaper [23].

Several studies reported the identification of pathogenic bacteria, such as *E. coli*, *Y. pestis*, and methicillin-resistant *S. aureus* strains, using bacteriophage amplification methodology, followed by detection of specific phage peptides by MALDI-TOF MS [191,192,193]. The use of LC-MS/MS for the detection of a lambda phage allowed the identification of *E. coli* contamination [23]. This method also allowed the identification of both, putative temperate and virulent phages, that were present in the bacterial strains analyzed.

Another advantage is that some of these novel methods do not require a phage amplification step (without the need for the pretreatment of bacterial lysis for bacteriophage replication) or bacterial culture, because the samples to be analyzed can be directly collected from the food or other materials they contaminate. These advantages considerably simplify the procedure, rendering it much cheaper and less time consuming. As reported above, these techniques can also detect temperate phages integrated in the host bacterial genome by identifying the proteins produced by the infected bacteria. They can also recognize additional phages that are infecting the host, as well as identifying not just the bacterial species, but also different bacterial strains [194,195]. The LC-ESI-MS/MS technique was successfully applied to identify peptides generated by a bacteriophage that infects 14 pathogenic strains of *Streptococcus* spp. (a bacterium that causes mastitis), that were detected as contaminants in milk. This discovery provided new insights into phage phylogenomics, as well as on the interactions between bacteriophages and the bacteria they infect. The analyses described above, involved tryptic digestion of *Streptococcus* peptides (100 μg of protein extracts) after cleaning through a C18 microSpinTM column, prior to being analyzed by LC-MS/MS. The resulting proteomic data were then processed by SEQUEST (Proteome Discoverer package, Thermo Fisher Scientific), and compared to the bacterial sequences stored in the UniProt/TrEMBL database. This MS method for the analysis and identification of peptides was performed in only 2–3 h, while the classical approach, requiring cell culture as well as protein extraction and purification, would have required 3 days. In addition, this MS approach allows the construction of phylogenetic trees, as the information obtained on *Streptococcus* spp. phage genomes, can be analyzed and compared, using available servers, like VICTOR (Virus Classification and Tree Building Online Resource). A total of 65 peptides were identified as specifically produced in *Streptococcus* bacteria, with peptides corresponding to proteins such as phage endopeptidases, phage repressors, uncharacterized phage polypeptides, and structural phage proteins. Therefore, the results obtained demonstrated that specific peptides are shared by a variety of closely related phages, as well as established a link between bacteriophage phylogeny and the host *Streptococcus* species. Moreover, the phage peptide M∗ATNLGQAYVQIM∗PSAK is unique and specific to *Streptococcus agalactiae* microorganisms. Taken together, these results establish the importance of diagnostic peptides, as they putatively represent major tools in the identification and characterization of pathogenic bacteria, such as the *Streptococcus* species that are responsible for mastitis [194].

The authors also applied the MS method mentioned above for the fast and accurate identification of 20 different *S. aureus* strains. In this case, they analyzed and characterized 79 peptides from bacteriophages infecting *S. aureus* strains, with 18 of the peptides being identified as specific to *S. aureus*. As bacteriophages are host-specific, these putative diagnostic peptides could play crucial roles as biomarkers for the detection and characterization of both *S. aureus* strains and *S. aureus* phages. As was the case for *Streptococcus,* see above, the data obtained for *Staphylococcus* also confirmed that specific peptides are shared by closely related phages. Furthermore, the *Staphylococcus* phages that share these peptides are closely related, as it is apparent in the phylogenetic tree [195]. Finally, we can conclude that proteomic analyses by LC-ESI-MS/MS provide significant insights into the origin of phages and play a relevant role as diagnostic peptide biomarkers.

A novel methodology, recently described, is based on the separation of phages by electromigration techniques, in combination with simultaneous proteome analyses, using laser desorption/ionization. Horka and colleagues, described the use of nano-etched fused-silica capillary, in combination with offline MALDI-TOF MS for the electrophoretic separation of bacteriophages found in large sample volumes, such as blood samples [196]. After electrophoretic analysis, the viability of the phages was determined, and the phage fractions were analyzed by MALDI-TOF MS. The same authors also studied the conditions required for the simultaneous separation and detection of both phage K1/420 and its *S. aureus* host by capillary isoelectric focusing (CIEF) and capillary zone electrophoresis (CZE) [197]. The bacteriophages were first purified, using preparative IEF, which increased their concentration by about 10-fold, prior to their detection using CZE and MALDI-TOF MS [197,198].

**Table 2:** Bacteriophage identification as a means to recognize pathogenic bacteria. This is a summary of relevant bacteriophages that have been analyzed by MS-related techniques, resulting in the identification of the pathogenic bacteria that harbored them.

Bacteriophages	Sample Source	Analytical Method	Reference
*Triaviruses*, *Phietaviruses*, *Biseptimaviruses*, *Kayviruses*, *Twortvirus*, P68virus	reference and isolates	MALDI-TOF MS	[193]
*Kayvirus* K1/420	medical isolate	CZE, MALDI-TOF MS	[197]
*Staphylococcal* phages (K1/420, 11, P68)	physiological saline solution, human serum	MALDI-TOF MS	[199]
*Staphylococcal* phages (K1/420, 11, P68, 3A, 77)	blood, serum	MALDI-TOF MS	[196]
*Yersinia pestis* phage ϕA1122 and *E. coli* phage MS2		MALDI-TOF MS	[191]
Methicillin-resistant *Staphylococcus aureus* phages		MALDI-TOF MS	[192]
*Streptococcus* spp. bacteriophages	Dairy products from mastitis	LC-ESI-MS/MS	[194]
*Staphylococcus* spp. bacteriophages	Dairy products from mastitis	LC-ESI-MS/MS	[195]
*E. coli* lamda phage		LC-ESI-MS/MS	[23]

## 5. Bacteriophage as Antimicrobials

Phages were discovered in the early 20th Century, due to their antibacterial activity. They were first administered to patients in Europe as antimicrobials to combat pathogenic strains of *Shigella* and *Salmonella* some years before the discovery of antibiotics. The lack of knowledge about phages together with the variable success obtained in their use as antimicrobials, prompted the health authorities of the time to abandon their use [1]. However, later, the main reason for the demise of bacteriophages was, undoubtedly, the discovery of antibiotics, widely heralded as all powerful ‘silver bullets’. However, the use of phages as treatment for bacterial infections continued in the Soviet Union, where they have been continually used since 1940, despite the fact that Western countries considered them unnecessary. It is only because of the threat posed by multidrug resistant bacteria, a current major hazard to world health that is rapidly and continually increasing due to the widespread use and misuse of antibiotics, that advanced Western countries are revisiting the antimicrobial utility of phages. Phage resurgence has opened the way for the use of these organisms in the treatment of bacterial infections, in humans and animals, as a single therapy or in combination with antibiotics [200]. The specificity of phages, that can only infect particular bacteria, represents a major advantage of phage therapy, as compared to conventional antimicrobials. This is one of the reasons for the interest generated by this therapy, which already have achieved successful outcomes, as reported both in Europe and the USA [201,202]. However, the safety and efficacy of phage therapy is still controversial, in the eyes of many health practitioners in Western countries [69]. Despite some drawbacks, research into this field is currently blooming, with many studies evaluating the efficacy of phages as biocontrol agents, in matters such as food and beverage contamination with pathogenic bacteria, as well as in biosanitization of equipment and work surfaces, directed to eradicate biofilms that could contaminate and shorten the shelf-life of foodstuffs.

Burrowes and colleagues [203] brought to light the “Appelmans protocol”, widely used in Eastern European countries to generate phages with novel lytic host ranges, which is achieved by recombination between the phages used in therapy. Phage encapsulation is one of the approaches developed to protect these organisms against harsh conditions, as well as to safeguard phage stability and, consequently, antimicrobial efficacy. González-Menéndez [204] and colleagues successful applied phage encapsulation in the food processing industry. Phage endolysins are currently the main proteins used as antibiotics, due to their ability to rapidly degrade the bacterial peptidoglycan cover which, in turn, results in cell death, both in Gram-positive and of Gram-negative bacteria. However, further research is required to ensure the safety and toxicity of this type of treatments [69].

All the data obtained to date from in vivo, ex vivo and in vitro phage therapy trials carried out in either humans or model animals to combat clinical multidrug-resistant (MDR) bacterial infections, indicate that this therapy provides significant protection against pathogenic bacteria. Moreover, studies on bio-preservation of food and beverages, as well as in bio-sanitization of surfaces, have demonstrated that phages produce significant bacterial growth suppression [205].

In fact, bacteriophages are currently being used in food products not only in the US, but also in Europe and Australia [206]. Indeed, some phage preparations have been approved for use in the USA, and are currently commercially available, including LISTEX P100; LMP-102^TM^, Listhield^TM^, ECP-100^TM^ (Ecoshield^TM^), SALMONELEX^TM^, AgriPhage^TM^, and Biophage-PA [205].

## 6. Bacteriophage as Vaccines

Vaccines are typically used against bacteria and viruses, both to avoid being infected by them and as prophylactic measures. Inactivated vaccines often require the administration of multiple dosses of dead pathogens to provide appropriate protection. On the other hand, attenuated vaccines, constructed by modification of live pathogens that render them no longer infectious, can provide effective protection without the need for multiple applications [60]. Phage display technology is a current technique that has proved useful in the identification of suitable phage elements, with the potential to increase the vaccines arsenal against infectious diseases. These elements could also play a major role in immune therapy to treat diseases such as cancer, due to the intrinsic ability of phages to display foreign antigens [207,208].

Typically, phage-based vaccines would contain a foreign antigen that by being fused to one of the bacteriophage capsid polypeptides will be displayed on the capsid surface. Another approach that involves attaching an antigen directly on the surface of the phage has the advantage of not altering the phage genome. Bacteriophage genomes can also be engineered to synthesize nucleic acid vaccines, rendering these organisms as putative vaccination vehicles that can target many cells. If the phages attach onto either MHC-I or MHC-II (MHC stands for major histocompatibility complex), they can produce both cytotoxic T lymphocytes (CTL) and an antibody-mediated response. Phage particles can also be taken up by antigen-presenting cells (APCs) that recognize foreign antigens [209]. Phages are currently considered safe for use in humans because they only infect prokaryotic cells. Additionally, bacteriophages replicate rapidly and uniformly, which makes them inexpensive and sustainable for large-scale production. Studies have been carried out on the use of phages in vaccines against foot and mouth disease [210], hepatitis B [211], and Epstein–Barr virus [212], as well as for several additional infectious diseases [1]. As mentioned above, phages are capable of inducing antigen presentation, by mechanisms involving both MHC-I and MHC-II, through a process known as cross-presentation. This represents an advantageous feature in the development of cancer immunotherapies, as CTLs activated by MHC-I recognition can kill tumor cells through the activation and interaction with PRRs (pattern recognition receptors), which causes the release of inflammatory cytokines that can modify the immunosuppressive environment surrounding the tumor. Vaccines based on phage display have been developed to target tumor cells such as those present in breast, liver, and lung cancers [59].

In conclusion, phage-based vaccines are designed to present antigens to the immune system, while generating the activation of stimulatory pathways such as those involving the adaptive immune system, for the purpose of generating CTLs and antibodies capable of binding to pathogens [209,213]. Furthermore, phage display technology allows the development of antibody-like drugs, hence overcoming the immunogenicity produced by these organisms that has limited their applications.

## 7. Concluding Remarks and Future Directions

The number of high-quality reports, based on phage assays, described in this review demonstrate the great potential displayed by phages, that could result in biotechnological applications beneficial to all humanity. Of particular interest is the use of a variety of phage-based techniques designed to identify both phages and their bacterial hosts, based on the recognition of specific phage proteins; this approach was demonstrated to detect low levels of viable bacteria in either food, water, or clinical samples.

Conventional culture-based and molecular methods designed for the detection of pathogenic bacteria are time consuming and labor intensive, but they remain as the main techniques currently in use. Alternative techniques, such as bacteriophage typing, used to be one the most employed methods to identify bacteria in complex sample matrices [51], with the ability to recognize individual bacterial strains. In addition, currently available techniques, such as phage display, permit the construction of libraries exhibiting the most suitable molecules to use for different purposes, as determined by in vitro panning [59]. Phage engineering, involving the construction of recombinant phages, allows the detection of a variety of live bacterial host cells present in many commercially important media, as they produce an easily detectable signal that can function as an indicator of bacterial viability [98]. Moreover, the use of bacteriophages, in combination with a variety of transducers, paved the way for the development of new biosensors and novel biomarkers that can be tailored for the specific detection of either molecules, such as proteins, or pathogenic microorganisms. This review particularly includes MS applications: novel MS techniques such as MALDI-TOF MS and LC-MS, used for the detection of phage proteins, that considerably reduce the time and labor required for the identification of bacteria.

At present, although multiple new technologies have been patented, there are just a few commercially available phage-based sensing devices (Ex. Corvium, In.) [50]. Undoubtedly, there is a great future for phage-based technologies as indicated by the current knowledge concerning the high variability and abundance of these organisms, and it can be predicted that further studies on this field will result in the development of novel phage-based biotechnological applications that will not only bring health benefits, but also improve commercial techniques [69]. Current research has already demonstrated that phages can play a crucial role in bio-sanitization, representing fast, economic tools, that can be used to identify microorganisms, present in either infections or as contaminants in clinical and food samples; future research will, unquestionably, extent the applications carried out by phages in the field of bio-sanitation. Another area in which phages can massively contribute to both human and animal health, is in the fight against antibiotic resistant bacteria, a major problem currently threatening humanity as a whole that is rapidly and continuous increasing, with multidrug resistant bacterial infections in humans becoming common all over the world. Phages are currently considered as one of the most promising alternatives for the treatment of multidrug-resistant bacterial infections, either on their own or in combination with antibiotics. Further putative alternative therapeutic approaches using phages include their use as vaccines against infectious disease and in immune therapy. Of particular interest is the phage encoded protein endolysin, that has already demonstrated its effectiveness as phage therapy in certain applications.

## Figures and Tables

**Table 1:** Summary of currently available therapeutic agents derived using phage display technology. Creative Commons Attribution license [59]. Abbreviations: TNFα: tumor necrosis factor-alpha, VEGFA: vascular endothelial growth factor A, BLyS: B-lymphocyte stimulator, *Bacillus anthracis* PA: *Bacillus anthracis* protective antigen, VEGFR2: vascular endothelial growth factor receptor 2, EGFR: epidermal growth factor receptor, IL-17A: interleukin-17A, PD-L1: programmed death-1 ligand-1, IL-23: interleukin-23, vWF: von Willebrand factor, IFNγ: interferon-gamma, pKal: plasma kallikrein, RA: rheumatoid arthritis, nAMD: neovascular age-related macular degeneration, SLE: systemic lupus erythematosus, GC: gastric carcinoma, NSCLC: non-small cell lung carcinoma, UC: urothelial carcinoma, MCC: Merkel cell carcinoma, aTTP: acquired thrombotic thrombocytopenic purpura, HLH: hemophagocytic lymphohistiocytosis, HCL: hairy cell leukemia, HAE: hereditary angioedema, Fab: fragment antigen-binding, scFv: single-chain variable fragment, CAT: Cambridge Antibody Technology.

Product Name	Nonproprietary Name	Target Antigen	First Application	Approved Year	Special Note on Phage Display Technology
Humira^®^	Adalimumab	TNFα	RA	2002	Humanization using guided selection method [74]
Lucentis^®^	Ranibizumab	VEGFA	nAMD	2006	*In vitro* affinity maturation [75]
Benlysta^®^	Belimumab	BLyS	SLE	2011	Isolation from CAT’s library (human naïve scFv library) [76]
ABthrax^®^	Raxibacumab	Bacillus anthracis PA	Inhaled anthrax	2012	Isolation from CAT’s library (human naïve scFv library) [77]
Cyramza^®^	Ramucirumab	VEGFR2	GC NSCLC	2014	Isolation from Dyax’s library (human naïve Fab library) [78]
Portrazza^®^	Necitumumab	EGFR	NSCLC	2015	Isolation from Dyax’s library (human naïve Fab library) [79]
Taltz^®^	Ixekizumab	IL-17A	Psoriasis	2016	Isolation from mouse immune Fab library [80]
Tecentriq^®^	Atezolizumab	PD-L1	UC NSCLC	2016	Isolation from Genentech’s library (human naïve library) [81,82]
Bavencio^®^	Avelumab	PD-L1	MCC	2017	Isolation from Dyax’s library (human naïve Fab library) [83]
Tremfya^®^	Guselkumab	IL-23	Psoriasis	2017	Isolation from HuCAL GOLD^®^ library (Synthetic Fab library) [84]
Cablivi^®^	Caplacizumab	vWF	aTTP	2018	Isolation from Camelidae-derived nanobody library [85]
Gamifant^®^	Emapalumab	IFNγ	HLH	2018	Isolation from CAT’s library (human naïve scFv library) [86]
Lumoxiti^®^	Moxetumomab pasudotox	CD22	HCL	2018	*In vitro* affinity maturation [87]
Takhzyro^®^	Lanadelumab	pKal	HAE	2018	Isolation from Dyax’s library (human naïve Fab library) [88]
